# Carcinomatous Tumour of the Neck Mistaken for an Aneurism of the External Carotid Artery

**Published:** 1844-10-19

**Authors:** 


### CARCINOMATOUS TUMOUR OF THE NECK MISTAKEN FOR AN ANEURISM OF THE EXTERNAL CAROTID ARTERY.

The following very instructive case, which lately occurred in Jervis-street Hospital, affords a most practical lessson of the great caution which should be observed in forming a diagnosis of the nature of all tumours, more particulariy those occurring in the neck:

John M'Manus, aged forty-four years, a labourer, of sallow, unhealthy appearance, was admitted into Jervis-street Hospital, July 5, 1844. There is an oval tumour about the size of a turkey's egg, situate on the right side of the neck, in that portion described as the post-superior triangle, penetrating deeply, so as to push the right tonsil forwards. A distinct pulsation can be felt in every portion of the tumour, which ceases when pressure is made on the carotid artery a little below the tumour; pressure on the artery does not diminish its size. It has a peculiar boggy inelastic feel; no discolouration of the skin covering it; pulse and respiration natural. The patient states it commenced about two years since, that it gradually increased in size until the present period, without causing him any annoyance whatever. The tumour being considered an aneurism of the external carotid artery, the operation of tying the common carotid artery in its first stage, was agreed on.

The operation was performed on the 20th July, in the usual manner, by Mr. O'Reily, assisted by Professors Harrison and Ellis, the other medical officers of the institution, and Mr. Adams, one of the surgeons to the Richmond Hospital, in the presence of a numerous class of students. The patient was put to bed after the operation, and ordered to take the following draught at night:—Acetate of opium, ten minims; water, one ounce. Make a draught.

21. The patients in the ward say he slept well during the night, that he awoke at two o'clock, A. M., and passed his urine. He appears to be now suffering from an attack of apoplexy; breathing stertorous; pulse slow and labouring; pupils dilated; extremities cold; he is perfectly insensible. Sinapisms applied to the abdomen and feet; ten ounces of blood from the back of the neck by cupping; cold effusion on the head. Under this treatment he partially recovered, but was attacked by a severe purging on the 25th. He lingered in this state until 29th July, when he died.

On examining the body the right carotid artery was found perfectly sound through its entire course, except where the ligature was applied. The tumour that in life so much resembled an aneurism, was found to be carcinomatous, part of which was lodged in the angle formed by the division of the common carotid artery. The brain was not examined. The tumour and the artery, with the ligature on it, are preserved at the hospital.—*Ibid.*

